# 3-(4-Chloro­anilino)-2,5-dimethyl­cyclo­hex-2-en-1-one

**DOI:** 10.1107/S1600536811005678

**Published:** 2011-04-29

**Authors:** Henry North, Kwame Wutoh, M’egya K. Odoom, Pradeep Karla, Kenneth R. Scott, Ray J. Butcher

**Affiliations:** a Department of Pharmaceutical Sciences, Howard University, 2300 4th Street NW, Washington, DC 20059, USA; b Bowie High School, Bowie, MD 20715, USA; c Fork Union Military Academy, Fork Union, VA 23055, USA; d Department of Chemistry, Howard University, 525 College Street NW, Washington, DC 20059, USA

## Abstract

In the title compound, C_14_H_16_ClNO, the dihedral angle between the benzene ring and the conjugated part of the cyclo­hexene ring is 61.7 (2)°. Part of the cyclo­hexene ring and one of the attached methyl groups are disordered over two orientations with occupancies of 0.602 (7) and 0.398 (7). In addition, the crystal studied was a racemic twin [Flack parameter = 0.58 (4)]. In the crystal, the mol­ecules are linked into chains in the *b*-axis direction by inter­molecular N—H⋯O hydrogen bonds. C—H⋯O and C—H⋯Cl inter­actions are also observed.

## Related literature

The title compound 3-(4-chloro­phenyl­amino)-2,5-dimethyl­cyclo­hex-2-enone possesses significant anti­convulsant properties. For the anti­convulsant properties of enamino­nes, see: Edafiogho *et al.* (1992 ▶); Eddington *et al.* (2003 ▶); Scott *et al.* (1993 ▶, 1995 ▶). For related structures see: Alexander *et al.* (2010 ▶, 2011 ▶).
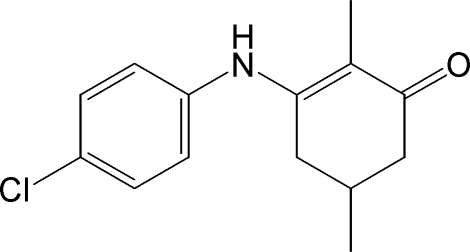


## Experimental

### 

#### Crystal data

C_14_H_16_ClNO
*M* *_r_* = 249.73
Monoclinic, 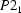

*a* = 6.0775 (5) Å
*b* = 8.8106 (5) Å
*c* = 12.5794 (7) Å
β = 99.904 (7)°
*V* = 663.5 (1) Å^3^
*Z* = 2
Cu *K*α radiation
μ = 2.41 mm^−1^
*T* = 295 K
0.45 × 0.28 × 0.10 mm

#### Data collection

Oxford Diffraction Xcalibur Ruby Gemini diffractometer
Absorption correction: multi-scan (*CrysAlis PRO*; Oxford Diffraction, 2009 ▶) *T*_min_ = 0.679, *T*_max_ = 1.000
2292 measured reflections
1705 independent reflections
1417 reflections with *I* > 2σ(*I*)
*R*_int_ = 0.029

#### Refinement

*R*[*F* ^2^ > 2σ(*F* ^2^)] = 0.054
*wR*(*F* ^2^) = 0.150
*S* = 1.00
1705 reflections
171 parameters
1 restraint
H-atom parameters constrained
Δρ_max_ = 0.22 e Å^−3^
Δρ_min_ = −0.17 e Å^−3^
Absolute structure: Flack (1983 ▶), 259 Friedel pairs
Flack parameter: 0.58 (4)

### 

Data collection: *CrysAlis PRO* (Oxford Diffraction, 2009 ▶); cell refinement: *CrysAlis PRO*; data reduction: *CrysAlis PRO*; program(s) used to solve structure: *SHELXS97* (Sheldrick, 2008 ▶); program(s) used to refine structure: *SHELXL97* (Sheldrick, 2008 ▶); molecular graphics: *SHELXTL* (Sheldrick, 2008 ▶); software used to prepare material for publication: *SHELXTL*.

## Supplementary Material

Crystal structure: contains datablocks I, global. DOI: 10.1107/S1600536811005678/hg2794sup1.cif

Structure factors: contains datablocks I. DOI: 10.1107/S1600536811005678/hg2794Isup2.hkl

Additional supplementary materials:  crystallographic information; 3D view; checkCIF report

## Figures and Tables

**Table 1:** Hydrogen-bond geometry (Å, °)

*D*—H⋯*A*	*D*—H	H⋯*A*	*D*⋯*A*	*D*—H⋯*A*
N1—H1⋯O1^i^	0.86	2.10	2.897 (4)	155
C6—H6*A*⋯O1^ii^	0.93	2.42	3.340 (4)	172
C12*A*—H12*A*⋯O1^ii^	0.97	2.58	3.439 (9)	147
C12*B*—H12*D*⋯Cl1^iii^	0.97	2.95	3.79 (3)	146

Symmetry codes: (i) 
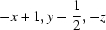
; (ii) 
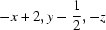
; (iii) 
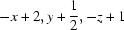
.

**Fig. 1.**
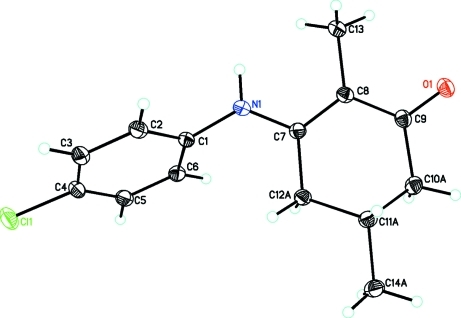
Diagram of 3-(4-Chlorophenylamino)-2,5-dimethylcyclohex-2-enone showing atom labeling scheme. Thermal ellipsoids drawn at the 30% probability level.

**Fig. 2.**
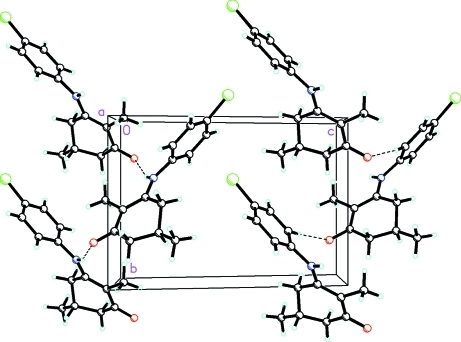
The molecular packing for 3-(4-Chlorophenylamino)-2,5-dimethylcyclohex-2-enone viewed down the a axis. Intermolecular interactions are shown by dashed lines.

## supplementary crystallographic information

### Comment

The study of enaminones has led to several compounds possessing anticonvulsant properties (Edafiogho *et al.*, 1992; Eddington *et al.*, 2003; Scott *et al.*, 1993, 1995; Alexander *et al.*, 2010, 2011). Our group has extensively studied the effects of modification of the enaminone with substitutions at the methyl ester, ethyl ester, and without the ester group. Early in our work, the N–H binding site was confirmed when it was found that no compound was active with the N–H proton missing. All of the active compounds were *para*-substituted with an electron-withdrawing group. A series of compounds with vinyl proton substitution has recently synthesized. The title compound, 3-(4-chlorophenylamino)-2,5-dimethylcyclohex-2-enone was exclusively active in the maximal electroshock seizure evaluation (MES) in mice, indicative of protection against tonic-clonic convulsions in humans. The MES test with mice revealed no activity at the 30 mg kg^-1^ dose, however in the 100 mg kg^-1^ dose, 3/3 of the animals were protected at 30 minutes and 0/3 of the animals were protected at 4 h. At a dose of 300 mg kg^-1^, 1/1 animals were protected 30 min and 4 h. There was toxicity at 30 min in 2/4 animals. In the rat MES study, at a dose of 30 mg kg^-1^, 1/4 of the animals were protected at 1 and 2 h with no toxicity.

Since the shape of the molecule is important in determining binding to the receptor sites it is of interest to note that the dihedral angle between the phenyl ring and the conjugated part of the cyclohexene ring is 61.7 (2)°. The backbone of the cyclohexene ring is disordered over two conformations with occupancies of 0.602 (7) and 0.398 (7), respectively. In addition the compound is a racemic twin (Flack parameter of 0.58 (4)). The molecules are linked in chains in the b direction by intermolecular N—H···O hydrogen bonds.

### Experimental

Iodomethane (11.2 ml, 0.18 mol, 1.5 equiv) was added to a solution of 5-methyl-1,3-cyclohexanedione (15.0 g, 0.119 mol) in 4 N aqueous sodium hydroxide (30 mL, 1.0 equiv of NaOH) in a two-neck 250 ml round bottom flask fitted with a magnetic stirrer and condenser. The solution was refluxed for 20 h and cooled to room temperature, then refrigerated at 0°C overnight. Vacuum filtration of the reaction mixture gave a crystalline mass dried to yield 9.24 g (54%). The crystalline mass, 2,5-dimethyl-1,3-cyclohexadione (2.10 g, 15 mmol), mp 170–172°C (lit. mp 130–131.5°C), 4-chloroaniline (2.32 g, 18 mmol), and toluene (60 ml) was added to a 150 ml single neck round bottom flask containing a stir bar. The solution was refluxed and stirred for 6 h with azeotropic removal of water by Dean-Stark trap. After standing overnight, crystals appeared. Evaporation under reduced pressure yielded crystals that were recrystallized from EtOAc, 44.3% yield (mp 183–185°C).

### Refinement

H atoms were placed in geometrically idealized positions and constrained to ride on their parent atoms with a C—H distance of 0.93 and 0.98 Å *U*_iso_(H) = 1.2*U*_eq_(C) and 0.96 Å for CH_3_ [*U*_iso_(H) = 1.5*U*_eq_(C)]. The H atoms attached to N were idealized with an N–H distance of 0.86 Å. The backbone of the cyclohexene was disordered over two conformations with occupancies of 0.602 (7) and 0.398 (7), respectively.

### Crystal data

**Table:**

C_14_H_16_ClNO	*F*(000) = 264
*M**_r_* = 249.73	*D*_x_ = 1.250 Mg m^−^^3^
Monoclinic, *P*2_1_	Cu *K*α radiation, λ = 1.54178 Å
Hall symbol: P 2yb	Cell parameters from 1339 reflections
*a* = 6.0775 (5) Å	θ = 5.0–73.9°
*b* = 8.8106 (5) Å	µ = 2.41 mm^−^^1^
*c* = 12.5794 (7) Å	*T* = 295 K
β = 99.904 (7)°	Chunk, colorless
*V* = 663.5 (1) Å^3^	0.45 × 0.28 × 0.10 mm
*Z* = 2	

### Data collection

**Table:**

Oxford Diffraction Xcalibur Ruby Gemini diffractometer	1705 independent reflections
Radiation source: Enhance (Cu) X-ray Source	1417 reflections with *I* > 2σ(*I*)
graphite	*R*_int_ = 0.029
Detector resolution: 10.5081 pixels mm^-1^	θ_max_ = 74.1°, θ_min_ = 6.2°
ω scans	*h* = −7→7
Absorption correction: multi-scan (*CrysAlis PRO*; Oxford Diffraction, 2009)	*k* = −10→5
*T*_min_ = 0.679, *T*_max_ = 1.000	*l* = −13→15
2292 measured reflections	

### Refinement

**Table:**

Refinement on *F*^2^	Secondary atom site location: difference Fourier map
Least-squares matrix: full	Hydrogen site location: inferred from neighbouring sites
*R*[*F*^2^ > 2σ(*F*^2^)] = 0.054	H-atom parameters constrained
*wR*(*F*^2^) = 0.150	*w* = 1/[σ^2^(*F*_o_^2^) + (0.1145*P*)^2^] where *P* = (*F*_o_^2^ + 2*F*_c_^2^)/3
*S* = 1.00	(Δ/σ)_max_ < 0.001
1705 reflections	Δρ_max_ = 0.22 e Å^−^^3^
171 parameters	Δρ_min_ = −0.17 e Å^−^^3^
1 restraint	Absolute structure: Flack (1983), 259 Friedel pairs
Primary atom site location: structure-invariant direct methods	Flack parameter: 0.58 (4)

### Special details

**Table:**

Geometry. All e.s.d.'s (except the e.s.d. in the dihedral angle between two l.s. planes) are estimated using the full covariance matrix. The cell e.s.d.'s are taken into account individually in the estimation of e.s.d.'s in distances, angles and torsion angles; correlations between e.s.d.'s in cell parameters are only used when they are defined by crystal symmetry. An approximate (isotropic) treatment of cell e.s.d.'s is used for estimating e.s.d.'s involving l.s. planes.
Refinement. Refinement of *F*^2^ against ALL reflections. The weighted *R*-factor *wR* and goodness of fit *S* are based on *F*^2^, conventional *R*-factors *R* are based on *F*, with *F* set to zero for negative *F*^2^. The threshold expression of *F*^2^ > σ(*F*^2^) is used only for calculating *R*-factors(gt) *etc*. and is not relevant to the choice of reflections for refinement. *R*-factors based on *F*^2^ are statistically about twice as large as those based on *F*, and *R*- factors based on ALL data will be even larger.

### Fractional atomic coordinates and isotropic or equivalent isotropic displacement parameters (Å^2^)

**Table:**

	*x*	*y*	*z*	*U*_iso_*/*U*_eq_	Occ. (<1)
Cl1	0.8577 (2)	−0.13354 (18)	0.49024 (9)	0.1059 (5)	
O1	0.7909 (4)	0.7372 (4)	−0.0704 (2)	0.0739 (7)	
N1	0.6375 (5)	0.3591 (5)	0.1708 (2)	0.0660 (7)	
H1	0.5033	0.3524	0.1357	0.079*	
C1	0.7018 (5)	0.2490 (4)	0.2532 (2)	0.0577 (8)	
C2	0.5554 (6)	0.2139 (6)	0.3223 (3)	0.0706 (10)	
H2A	0.4243	0.2693	0.3193	0.085*	
C3	0.6030 (7)	0.0966 (6)	0.3961 (3)	0.0775 (11)	
H3A	0.5036	0.0717	0.4419	0.093*	
C4	0.7978 (7)	0.0183 (5)	0.4006 (3)	0.0716 (10)	
C5	0.9464 (6)	0.0537 (5)	0.3342 (3)	0.0685 (9)	
H5A	1.0803	0.0009	0.3397	0.082*	
C6	0.8969 (6)	0.1680 (5)	0.2591 (3)	0.0630 (8)	
H6A	0.9957	0.1904	0.2125	0.076*	
C7	0.7639 (6)	0.4728 (4)	0.1416 (3)	0.0576 (8)	
C8	0.7074 (6)	0.5456 (4)	0.0452 (3)	0.0576 (7)	
C9	0.8352 (5)	0.6705 (5)	0.0176 (3)	0.0614 (8)	
C10A	1.051 (3)	0.719 (3)	0.0899 (17)	0.066 (3)	0.602 (7)
H10A	1.0710	0.8274	0.0847	0.080*	0.602 (7)
H10B	1.1773	0.6689	0.0664	0.080*	0.602 (7)
C11A	1.0433 (10)	0.6749 (8)	0.2071 (5)	0.0654 (13)	0.602 (7)
H11A	0.9295	0.7376	0.2324	0.078*	0.602 (7)
C12A	0.977 (3)	0.5058 (16)	0.2158 (13)	0.059 (2)	0.602 (7)
H12A	1.0945	0.4414	0.1976	0.070*	0.602 (7)
H12B	0.9599	0.4834	0.2894	0.070*	0.602 (7)
C14A	1.270 (3)	0.708 (2)	0.279 (2)	0.081 (4)	0.602 (7)
H14A	1.3132	0.8111	0.2684	0.122*	0.602 (7)
H14B	1.3813	0.6404	0.2606	0.122*	0.602 (7)
H14C	1.2575	0.6937	0.3535	0.122*	0.602 (7)
C10B	1.015 (6)	0.732 (5)	0.110 (3)	0.066 (3)	0.398 (7)
H10C	1.1249	0.7883	0.0783	0.080*	0.398 (7)
H10D	0.9443	0.8023	0.1529	0.080*	0.398 (7)
C11B	1.1314 (16)	0.6105 (13)	0.1820 (8)	0.0654 (13)	0.398 (7)
H11B	1.1935	0.5344	0.1386	0.078*	0.398 (7)
C12B	0.950 (5)	0.540 (3)	0.233 (2)	0.059 (2)	0.398 (7)
H12C	0.8849	0.6157	0.2745	0.070*	0.398 (7)
H12D	1.0119	0.4596	0.2822	0.070*	0.398 (7)
C14B	1.321 (6)	0.673 (4)	0.272 (4)	0.081 (4)	0.398 (7)
H14D	1.4200	0.5920	0.3000	0.122*	0.398 (7)
H14E	1.2559	0.7160	0.3298	0.122*	0.398 (7)
H14F	1.4044	0.7502	0.2423	0.122*	0.398 (7)
C13	0.5116 (7)	0.4931 (5)	−0.0383 (3)	0.0716 (10)	
H13A	0.5080	0.3842	−0.0402	0.107*	
H13B	0.5279	0.5312	−0.1080	0.107*	
H13C	0.3749	0.5306	−0.0196	0.107*	

### Atomic displacement parameters (Å^2^)

**Table:**

	*U*^11^	*U*^22^	*U*^33^	*U*^12^	*U*^13^	*U*^23^
Cl1	0.1398 (11)	0.0944 (8)	0.0775 (6)	0.0012 (8)	0.0015 (6)	0.0320 (6)
O1	0.0719 (15)	0.0806 (17)	0.0706 (14)	0.0063 (14)	0.0158 (11)	0.0202 (13)
N1	0.0611 (14)	0.0737 (18)	0.0575 (14)	−0.0048 (16)	−0.0060 (11)	0.0102 (15)
C1	0.0651 (18)	0.0592 (19)	0.0451 (14)	−0.0076 (16)	−0.0009 (12)	0.0016 (13)
C2	0.0615 (18)	0.085 (3)	0.0630 (18)	−0.0002 (19)	0.0042 (14)	0.0081 (18)
C3	0.074 (2)	0.100 (3)	0.0582 (17)	−0.009 (2)	0.0091 (15)	0.015 (2)
C4	0.087 (3)	0.068 (2)	0.0535 (17)	−0.008 (2)	−0.0037 (15)	0.0098 (16)
C5	0.074 (2)	0.065 (2)	0.0623 (18)	0.003 (2)	−0.0017 (15)	−0.0009 (17)
C6	0.0663 (18)	0.066 (2)	0.0556 (15)	−0.0063 (19)	0.0059 (13)	−0.0038 (16)
C7	0.0562 (16)	0.0566 (19)	0.0579 (16)	−0.0011 (15)	0.0042 (12)	−0.0010 (15)
C8	0.0563 (16)	0.0581 (18)	0.0566 (16)	0.0059 (16)	0.0046 (12)	−0.0017 (15)
C9	0.0585 (16)	0.0611 (19)	0.0660 (18)	0.0088 (17)	0.0144 (14)	0.0059 (17)
C10A	0.057 (7)	0.072 (6)	0.073 (8)	−0.005 (5)	0.021 (4)	0.005 (6)
C11A	0.062 (3)	0.060 (4)	0.070 (3)	0.000 (3)	0.001 (2)	−0.007 (3)
C12A	0.065 (5)	0.051 (7)	0.056 (6)	0.003 (5)	0.000 (3)	−0.006 (4)
C14A	0.070 (10)	0.075 (10)	0.092 (4)	−0.006 (6)	−0.007 (6)	−0.005 (7)
C10B	0.057 (7)	0.072 (6)	0.073 (8)	−0.005 (5)	0.021 (4)	0.005 (6)
C11B	0.062 (3)	0.060 (4)	0.070 (3)	0.000 (3)	0.001 (2)	−0.007 (3)
C12B	0.065 (5)	0.051 (7)	0.056 (6)	0.003 (5)	0.000 (3)	−0.006 (4)
C14B	0.070 (10)	0.075 (10)	0.092 (4)	−0.006 (6)	−0.007 (6)	−0.005 (7)
C13	0.072 (2)	0.075 (2)	0.0617 (19)	0.000 (2)	−0.0045 (16)	0.0077 (18)

### Geometric parameters (Å, °)

**Table:**

Cl1—C4	1.747 (4)	C10A—H10B	0.9700
O1—C9	1.241 (4)	C11A—C14A	1.54 (2)
N1—C7	1.351 (5)	C11A—C12A	1.552 (18)
N1—C1	1.424 (5)	C11A—H11A	0.9800
N1—H1	0.8600	C12A—H12A	0.9700
C1—C6	1.375 (5)	C12A—H12B	0.9700
C1—C2	1.382 (5)	C14A—H14A	0.9600
C2—C3	1.386 (6)	C14A—H14B	0.9600
C2—H2A	0.9300	C14A—H14C	0.9600
C3—C4	1.363 (6)	C10B—C11B	1.50 (4)
C3—H3A	0.9300	C10B—H10C	0.9700
C4—C5	1.367 (5)	C10B—H10D	0.9700
C5—C6	1.378 (6)	C11B—C12B	1.50 (3)
C5—H5A	0.9300	C11B—C14B	1.58 (5)
C6—H6A	0.9300	C11B—H11B	0.9800
C7—C8	1.363 (5)	C12B—H12C	0.9700
C7—C12A	1.489 (19)	C12B—H12D	0.9700
C7—C12B	1.58 (3)	C14B—H14D	0.9600
C8—C9	1.424 (5)	C14B—H14E	0.9600
C8—C13	1.518 (5)	C14B—H14F	0.9600
C9—C10A	1.52 (3)	C13—H13A	0.9600
C9—C10B	1.55 (4)	C13—H13B	0.9600
C10A—C11A	1.53 (2)	C13—H13C	0.9600
C10A—H10A	0.9700		
			
C7—N1—C1	127.3 (3)	C10A—C11A—C14A	110.2 (14)
C7—N1—H1	116.4	C10A—C11A—C12A	111.1 (13)
C1—N1—H1	116.4	C14A—C11A—C12A	110.9 (12)
C6—C1—C2	119.5 (3)	C10A—C11A—H11A	108.2
C6—C1—N1	121.3 (3)	C14A—C11A—H11A	108.2
C2—C1—N1	119.0 (3)	C12A—C11A—H11A	108.2
C1—C2—C3	120.4 (4)	C7—C12A—C11A	110.6 (11)
C1—C2—H2A	119.8	C7—C12A—H12A	109.5
C3—C2—H2A	119.8	C11A—C12A—H12A	109.5
C4—C3—C2	119.0 (4)	C7—C12A—H12B	109.5
C4—C3—H3A	120.5	C11A—C12A—H12B	109.5
C2—C3—H3A	120.5	H12A—C12A—H12B	108.1
C3—C4—C5	121.3 (4)	C11B—C10B—C9	114 (3)
C3—C4—Cl1	119.8 (3)	C11B—C10B—H10C	108.8
C5—C4—Cl1	118.9 (3)	C9—C10B—H10C	108.8
C4—C5—C6	119.8 (4)	C11B—C10B—H10D	108.8
C4—C5—H5A	120.1	C9—C10B—H10D	108.8
C6—C5—H5A	120.1	H10C—C10B—H10D	107.7
C1—C6—C5	120.1 (3)	C10B—C11B—C12B	104.5 (18)
C1—C6—H6A	120.0	C10B—C11B—C14B	113 (2)
C5—C6—H6A	120.0	C12B—C11B—C14B	110 (2)
N1—C7—C8	121.5 (3)	C10B—C11B—H11B	109.7
N1—C7—C12A	116.6 (8)	C12B—C11B—H11B	109.7
C8—C7—C12A	121.7 (8)	C14B—C11B—H11B	109.7
N1—C7—C12B	116.5 (12)	C11B—C12B—C7	109.0 (17)
C8—C7—C12B	120.8 (12)	C11B—C12B—H12C	109.9
C12A—C7—C12B	15.4 (10)	C7—C12B—H12C	109.9
C7—C8—C9	121.1 (3)	C11B—C12B—H12D	109.9
C7—C8—C13	121.3 (3)	C7—C12B—H12D	109.9
C9—C8—C13	117.6 (3)	H12C—C12B—H12D	108.3
O1—C9—C8	122.6 (3)	C11B—C14B—H14D	109.5
O1—C9—C10A	115.7 (10)	C11B—C14B—H14E	109.5
C8—C9—C10A	121.3 (10)	H14D—C14B—H14E	109.5
O1—C9—C10B	121.3 (16)	C11B—C14B—H14F	109.5
C8—C9—C10B	115.6 (15)	H14D—C14B—H14F	109.5
C10A—C9—C10B	14.3 (11)	H14E—C14B—H14F	109.5
C9—C10A—C11A	109.7 (13)	C8—C13—H13A	109.5
C9—C10A—H10A	109.7	C8—C13—H13B	109.5
C11A—C10A—H10A	109.7	H13A—C13—H13B	109.5
C9—C10A—H10B	109.7	C8—C13—H13C	109.5
C11A—C10A—H10B	109.7	H13A—C13—H13C	109.5
H10A—C10A—H10B	108.2	H13B—C13—H13C	109.5
			
C7—N1—C1—C6	−49.7 (6)	C13—C8—C9—C10A	−172.1 (8)
C7—N1—C1—C2	136.0 (4)	C7—C8—C9—C10B	−9.3 (13)
C6—C1—C2—C3	−1.0 (6)	C13—C8—C9—C10B	173.0 (13)
N1—C1—C2—C3	173.4 (4)	O1—C9—C10A—C11A	159.1 (10)
C1—C2—C3—C4	1.1 (6)	C8—C9—C10A—C11A	−27.6 (17)
C2—C3—C4—C5	0.3 (6)	C10B—C9—C10A—C11A	43 (10)
C2—C3—C4—Cl1	−178.1 (3)	C9—C10A—C11A—C14A	174.3 (12)
C3—C4—C5—C6	−1.7 (6)	C9—C10A—C11A—C12A	50.9 (17)
Cl1—C4—C5—C6	176.7 (3)	N1—C7—C12A—C11A	−152.2 (7)
C2—C1—C6—C5	−0.5 (5)	C8—C7—C12A—C11A	32.7 (10)
N1—C1—C6—C5	−174.8 (3)	C12B—C7—C12A—C11A	−59 (6)
C4—C5—C6—C1	1.9 (6)	C10A—C11A—C12A—C7	−54.1 (13)
C1—N1—C7—C8	162.5 (3)	C14A—C11A—C12A—C7	−177.0 (10)
C1—N1—C7—C12A	−12.5 (7)	O1—C9—C10B—C11B	−149.8 (12)
C1—N1—C7—C12B	−29.8 (10)	C8—C9—C10B—C11B	37.9 (17)
N1—C7—C8—C9	176.7 (3)	C10A—C9—C10B—C11B	−79 (10)
C12A—C7—C8—C9	−8.4 (7)	C9—C10B—C11B—C12B	−63.3 (18)
C12B—C7—C8—C9	9.6 (10)	C9—C10B—C11B—C14B	177.3 (18)
N1—C7—C8—C13	−5.6 (6)	C10B—C11B—C12B—C7	60 (2)
C12A—C7—C8—C13	169.2 (5)	C14B—C11B—C12B—C7	−178.3 (16)
C12B—C7—C8—C13	−172.8 (9)	N1—C7—C12B—C11B	155.6 (11)
C7—C8—C9—O1	178.5 (4)	C8—C7—C12B—C11B	−36.6 (17)
C13—C8—C9—O1	0.7 (5)	C12A—C7—C12B—C11B	61 (6)
C7—C8—C9—C10A	5.6 (9)		

### Hydrogen-bond geometry (Å, °)

**Table:**

*D*—H···*A*	*D*—H	H···*A*	*D*···*A*	*D*—H···*A*
N1—H1···O1^i^	0.86	2.10	2.897 (4)	155
C6—H6A···O1^ii^	0.93	2.42	3.340 (4)	172
C12A—H12A···O1^ii^	0.97	2.58	3.439 (9)	147
C12B—H12D···Cl1^iii^	0.97	2.95	3.79 (3)	146

Symmetry codes: (i) −*x*+1, *y*−1/2, −*z*; (ii) −*x*+2, *y*−1/2, −*z*; (iii) −*x*+2, *y*+1/2, −*z*+1.
